# PROUD: Effects of preoperative long-term immunonutrition in patients listed for liver transplantation

**DOI:** 10.1186/1745-6215-8-20

**Published:** 2007-08-27

**Authors:** Arash Nickkholgh, Heinz Schneider, Jens Encke, Markus W Büchler, Jan Schmidt, Peter Schemmer

**Affiliations:** 1 Department of General Surgery, Ruprecht-Karls University, Heidelberg, Germany; 2 HealthEcon AG, Basel, Switzerland; 3 Department of Internal Medicine, Ruprecht-Karls University, Heidelberg, Germany

## Abstract

**Background:**

Patients with end stage liver disease are characteristically malnourished which is associated with poor outcome. Formulas enriched with arginine, ω-3 fatty acids, and nucleotides, "immunonutrients", potentially improve their nutritional status. This study is designed to evaluate the clinical outcome of long-term "immunonutrition" of patients with end-stage liver disease while on the waiting list for liver transplantation.

**Methods/design:**

A randomized controlled double blind multi-center clinical trial with two parallel groups comprising a total of 142 newly registered patients for primary liver transplantation has been designed to assess the safety and efficacy of the long-term administration of ORAL IMPACT^®^, an "immunonutrient" formula, while waiting for a graft. Patients will be enrolled the day of registration on the waiting list for liver transplantation. Study ends on the day of transplantation. Primary endpoints include improved patients' nutritional and physiological status, as measured by mid-arm muscle area, triceps skin fold thickness, grip strength, and fatigue score, as well as patients' health related quality of life. Furthermore, patients will be followed for 12 postoperative weeks to evaluate anabolic recovery after transplantation as shown by reduced post-transplant mechanical ventilation, hospital stay, wound healing, infectious morbidities (pneumonia, intraabdominal abscess, sepsis, line sepsis, wound infection, and urinary tract infection), acute and chronic rejection, and mortality.

**Discussion:**

Formulas enriched with arginine, ω-3 fatty acids, and nucleotides have been proven to be beneficial in reducing postoperative infectious complications and length of hospital stay among the patients undergoing elective gastrointestinal surgery. Possible mechanisms include downregulation of the inflammatory responses to surgery and immune modulation rather than a sole nutritional effect.

**Trial registration:**

ClinicalTrials.gov NCT00495859

## Background

### Immunonutrition

Patients with end-stage liver disease (ESLD) characteristically suffer from protein-energy malnutrition (PEM) and hypermetabolism due to abnormal nutrient and caloric intake, decreased intestinal absorption, and metabolic disturbances, which is associated with poor outcome including poor perceived health-related quality of life (HRQL) [1,2] and unsatisfactory postoperative course, i.e., prolonged intensive care unit and total hospital stay, prolonged mechanical ventilation, and increased morbidities and mortality [3,4]. The biochemical nutritional derangements include deficiency in ω-3 and ω-6 long-chain fatty acids [5], insulin resistance [6], growth hormone resistance [7,8], increased proinflammatory cytokines (e.g., TNF-α, IL-1, IL-6) [9], and increased lipid peroxidation due to a deficiency in endogenous antioxidants, i.e. vitamin E and carotenoids [10,11], all of which lead to a clinical manifestation of progressive lipolysis and proteolysis. Supplementation with ω-3 fatty acids may downregulate proinflammatory cytokine production and modulate eicosanoid synthesis [12-14]. The latter molecules lead to the production of vasodilators, i.e. LTB5, PGE3, PGI3, and TxA3, thus acting against inflammation, aggregation, adhesion, and chemotaxis [15]. Arginine, which cannot be produced in catabolic states, stimulates the release of anabolic hormones (growth hormone, insulin), improves nitrogen balance and wound healing, upregulates immune function, and enhances NO biosynthesis [16-18]. An increase in protein synthesis can be achieved with nucleotides [19]. Nucleotides can also enhance a variety of host defense mechanisms [20]. Formulas enriched with arginine, ω-3 fatty acids, and nucleotides (immune enhancing diets or the so-called "immunonutrients" – which are designed to modulate a patient's immune response and can hence augment basic nutritional repletion) have been proven to be beneficial in reducing postoperative infectious complications and length of hospital stay among the patients undergoing elective gastrointestinal surgery, especially in those who are malnourished preoperatively [21]. Possible mechanisms include downregulation of the inflammatory responses to surgery and immune modulation rather than a sole nutritional effect.

### Trial Rationale

The interval between listing and transplantation, which may usually extend to several months, provides a unique opportunity for the physician to institute a nutritional therapy. However, clinical studies concerning the long-term preoperative supplementation with such immune-modulating formulas in the field of transplant surgery are rare and inconclusive. Up to 30 papers between 1995 and 2005 under the topic of "immunonutrition and surgery" were found in the literature and reviewed. However, none investigated the long-term preoperative outcome of such supplementation (all just covered a short-term peri- or postoperative period), and few, if any, directly considered the liver transplant patients. Concerning the supplementation of the patients in the waiting list for transplantation, 4 studies were found in the literature, of which only one was randomized. The latter trial investigated the preoperative nutritional supplementation in patients awaiting elective orthotopic liver transplantation (LT) and was designed to determine whether nutritional supplementation improves nutritional status and affects outcome after transplantation. However, it failed to reveal any significant effect on outcome after transplantation, although it improved some parameters of nutritional status pretransplantation. The investigators didn't use any immune-enhancing diet in the trial [22].

Preliminary results of a recent nonrandomized pilot study suggest that immunonutrition may improve short-term preoperative nutritional status, hasten recovery after transplantation, and reduce postoperative infectious complications [23]. Preoperative feeding was started after patients got to the top of the waiting list (and was continued for at least 5 days posttransplant). ORAL IMPACT^® ^(Novartis Consumer Health, Nyon, Switzerland) was used with no adverse short-term consequences.

Therefore, this study is unique because it is randomized and it fully investigates the effects of ORAL IMPACT^® ^during a long-term period in patients waiting for a LT. In this manner, the comparison between patients receiving ORAL IMPACT^® ^(arginine/ω-3 fatty acids/nucleotide enriched formula) or an isocaloric isonitrogenous control may help to identify the exact role of immunonutrition in patients with ESLD.

## Methods/Design

### Subject recruitment

After the positive vote of the ethics committee of the Faculty of Medicine, Ruprecht-Karls University of Heidelberg, enrollment of 142 newly registered patients for primary LT will be started early in September 2007 in this prospective multicenter placebo-controlled randomized double-blind clinical trial with two parallel treatment groups receiving either study product or control supplement. Patients are considered for recruitment to the study according to inclusion and exclusion criteria (Table 1). Each subject is expected to be recruited on the day of registration on the waiting list for LT. Study nutrition ends on the day of transplantation. To assess early outcome after transplantation patients are followed for 12 postoperative weeks. The duration of the overall trial is expected to last approximately 36 months. The actual duration of trial may vary due to the availability of patients meeting the criteria after registration for LT.

**Table 1:** Criteria for inclusion and exclusion of patients.

**Inclusion criteria**	**Exclusion criteria**
patients meeting all of the following criteria are considered for inclusion in the study: - men and women, 18–68 years of age - scheduled for first liver transplantation - written informed consent - protein-calorie malnutrition defined as mid-arm muscle area (MAMA) <85% standard	patients with any of the following will not be included in the trial: - patients <18 and >68 years - pregnant or nursing women - history of hypersensitivity to arginine, ω-3 fatty acids, or nucleotides - inability to take oral nutrition - patients with fulminant or subacute hepatic failure - mental condition rendering the subject incapable of understanding the nature, scope, and consequences of the trial No subject will be enrolled in this study more than once.

### Objectives and endpoints

Primary objective is to determine the safety and efficacy of the long-term administration of immunonutrients, and assessment of the patients' nutritional and physiological status as well as quality of life, while waiting for a graft. Secondary Objective is to investigate the effects of immunonutrition on post-transplant morbidities.

### Trial design and schedule (Fig. 1)

**Figure 1:**
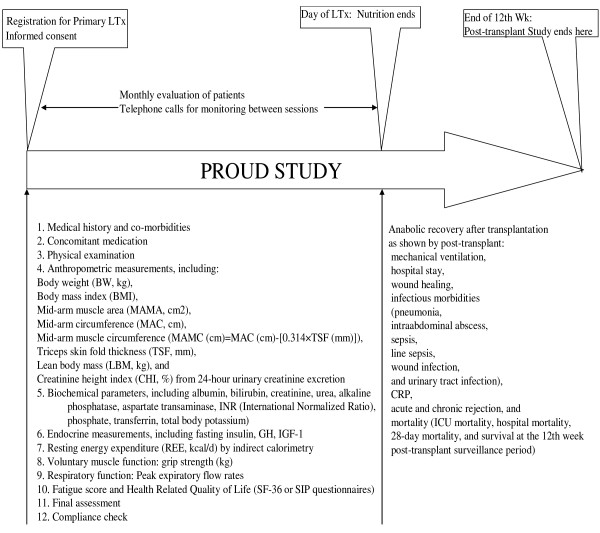
**Scheme depicting the workflow of the study**. Each subject will be recruited on the day of registration on the waiting list for liver transplantation after giving informed consent. Study nutrition ends on the day of transplantation. Subjects are randomized to two parallel groups treated for study or control product. Furthermore, patients will be followed for 12 weeks after transplantation.

Site number, patients' randomization number and initials, all demographic data (date of birth, sex, height, weight), medical history (including a complete dietary history and MELD score), physical examination, comorbidities, and concomitant medications are documented on day one. Furthermore, anthropometric, physiologic, and biochemical measurements are carried out and documented. The aforementioned measurements are performed during monthly visits until transplantation. The patients consume either the study product (ORAL IMPACT^®^) (Table 2) or control product (IMPACT-control supplement) (Table 2) once a day for the period of waiting until a graft is available. During each visit, the following data are recorded in the electronic case report form (eCRF):

**Table 2:** Composition of ORAL IMPACT^® †^and IMPACT Control supplement (in powder form)

Content (1 Sachet)		**ORAL IMPACT^® ^**(74 g)	**IMPACT Control **(74 g)
-Protein	g	16.7	13.0
-Casein/Whey	g	(13)	(13)
-L Arginine	g	(3.74)	-
-Fat	g	8.3	8.3
-Essential FA (LA+LNA)	g	(0.94)	(2.6)
-n-6-FA (LA)	g	(0.74)	(2.6)
-n-3FA (EPA+DHA+LNA)	g	(1)	-
Carbohydrates	g	40.2	43.94
Fibres	g	3	3
RNA	mg	390	-
Energy	g	302.8	302.8
-kcal/ml		§	§
-P:F:C	%	22:25:53	17:25:58
Sodium	mg	320	320
Potassium	mg	402	402
Calcium	mg	240	240
Magnesium	mg	80	80
Phosphorus	mg	216	216
Chloride	mg	480	480
Iron	mg	3.6	3.6
Copper	mg	0.5	0.5
Manganese	mg	0.6	0.6
Zinc	mg	4.5	4.5
Fluoride	mg	0.5	0.5
Iodine	mcg	45	45
Chromium	mcg	30	30
Molybdenum	mcg	60	60
Selenium	mcg	14	14
Retinol (Vit. A.)	mg	0.3	0.3
Calciferol (Vit. D)	mcg	2	2
Tocopherol (Vit. E)	mg	4	4
Phyllochin. (Vit. K1)	mg	20	20
Thiamine (Vit. B1)	mg	0.36	0.36
Riboflavine (Vit. B2)	mg	0.52	0.52
Pyridoxine (Vit. B6)	mg	0.44	0.44
Cyanocobal. (Vit. B12)	mcg	1.2	1.2
Ascorbic Acid (Vit. C)	mg	20	20
Biotin	mcg	30	30
Folic Acid	mcg	60	60
Niacinamide	mg	4.8	4.8
Pantothenic Acid	mg	2.4	2.4

^†^; IMPACT^® ^is a food for special medical purpose (FSMP)

§ content of sachet to be dissolved in 250 ml water for each serving

FA; fatty acid, LA; linoleic acid, LNA; linolenic acid, EPA; Eicosapentaenoic acid, DHA; docosahexaenoic acid, RNA; ribonucleic acid, kcal; kilocalorie, ml; millilitre, P:F:C; protein:fat: carbohydrate, Vit.; vitamin.

Reference:

A. Anthropometric measurements, including:

• Body weight (BW, kg),

• Body mass index (BMI),

• Mid-arm muscle area (MAMA, cm2)

• Mid-arm circumference (MAC, cm),

• Mid-arm muscle circumference (MAMC (cm) = MAC (cm) - [0.314 × TSF (mm)]),

• Triceps skinfold thickness (TSF, mm),

• Lean body mass (LBM, kg), and

• Creatinine height index (CHI, %) from 24-hour urinary creatinine excretion (patients are advised to collect their urine for 24 hours right before each monthly visit and bring it to the center with them)

B. Biochemical parameters, including albumin, bilirubin, creatinine, urea, alkaline phosphatase, transaminases (ALT, AST), INR, phosphate, transferrin, total body potassium)

C. Endocrine measurements, including fasting insulin, GH, and IGF-1

D. Resting energy expenditure (REE, kcal/d) by indirect calorimetry

E. Voluntary muscle function: grip strength (kg)

F. Respiratory function: Peak expiratory flow rates

G. Fatigue score and HRQL (SF-36 questionnaires [24]).

During each monthly visit, patients will be provided with the required monthly amount of study product (or control product) and are asked to return the emptied study boxes with them to the next monthly session to warrant that they are compliant with the trial regimen. Further, between the two consecutive monthly visits, the trial investigator has to phone call the patient weekly to check for both compliance and adverse events. Deviations of the protocol are documented in the eCRF. The trial ends on the day a graft becomes available. A patient consuming a total of 80% of the calories prescribed over the entire preoperative study period is considered a "completer". Post-transplant investigations concern anabolic recovery after transplantation as shown by:

• post-transplant mechanical ventilation

• total hospital stay

• wound healing

• infectious morbidities (pneumonia, intraabdominal abscess, sepsis, central line sepsis, wound infection, and urinary tract infection)

• CRP levels

• acute and chronic rejection, and

• mortality (ICU mortality, hospital mortality, 28-day mortality, and survival at the 12^th ^week post-transplant surveillance period).

These post-transplant parameters are investigated and documented during the patients' posttransplant hospital stay daily and further until the 12^th ^week posttransplant through postoperative follow-up sessions. Sepsis is defined as the presence of at least 2 out of the following 4 criteria: (1) temperature >38°C or <36°C, (2) tachycardia >90/min, (3) leukocytosis >12/nl or leukopenia <4/nl, and (4) respiratory insufficiency (any of the 3 following criteria: respiratory rate >20/min, hyperventilation with PaCO_2 _<32 mmHg or PaO_2 _<70 mmHg (spontaneous breathing) or PaO_2_/FiO_2 _<175 mmHg (mechanical ventilation) [25]. CRP will be measured weekly posttransplant until the patient is discharged, and then along with other investigations through postoperative follow-up sessions. During the posttransplant phase, subjects will routinely receive immunosuppressive therapy which includes cyclosporine A, mycophenolate (Myfortic^®^) and steroids. The immunosuppression will be done with or without induction therapy [Simulect^® ^(Basiliximab)].

### Sample size calculation

The sample size calculation is based on the detection of significant differences in MAMA, one of the primary endpoint parameters of this trial. A clinical trial on enteral hyperalimentation in malnourished patients with cirrhosis and ascites [26] was used as an exemplary model. The rate was measured in percent per day, i.e. MAMA changes divided by number of days between admission and discharge. After eliminating the gross outlier of this study, the standard deviation of this rate was 0.21% per day. The clinically relevant difference was set to 0.1% MAMA per day. Therefore, the sample size necessary for the trial with a power of 80% and a two-sided significance level of 0.05 was calculated to be 71 patients per group. An assumed 10% drop-out rate in this trial (due to non-compliance, intolerance, premature discontinuation, etc) will raise the sample size to 78 patients per group. Therefore, at least a total of 156 patients have to be included to the trial in order to yield 142 completers.

### Randomization and treatment

Randomization (1:1) will be performed by the algorithm programmed in specific computer software and a randomization list will be prepared. The randomization list will be prepared by the institution that is responsible for the packaging of the study and control products and kept in safe and confidential custody at this institution. Both study product and control product will be of same form and appearance (powder) including the packaging material. If it is medically imperative to know which product the patient consumes, emergency envelopes contain the information on the subject's study product.

### Adverse events

All adverse events (AE) are recorded. Events related to the initial diagnosis for LT, to the transplantation procedure itself, or problems associated with routine procedures after transplantation, e.g., liver biopsy, are not to be noted as AE or serious adverse event (SAE) unless the investigator deems the events to be a cause of the study product. All SAE potentially associated with the application of study product must be documented on a SAE form which has to be sent to the principal investigator within 24 hours or latest on the following working day. The principal investigator ensures that SAE are reported to the safety board, ethics committee, and to further investigators, if applicable.

### Quality assurance

The study is performed according to the principles of the ICH-GCP consolidated guidelines as required by regulatory agencies [27] and the ethical principles according to the current revision of the Declaration of Helsinki [28] and local legal and regulatory requirements. The trial is monitored by HealthEcon AG according to standard operational procedures that is based on ICH-GCP guidelines.

An independent safety board monitors closely the proper conduct of the trial and all SAE reports to ensure the safety of the subjects during the course of the study.

### Statistics and data management

All analyses will be carried out on an "intent-to-treat" basis. However, attempts will be made to analyze "per protocol", "completer", and "intent-to-treat" populations separately, when statistically appropriate. Repeated-measures analysis of variance (ANOVA) will be used followed by t-test when significant differences are detected by ANOVA. Pearson Chi-square test or Fisher's exact test will be used when appropriate. Times to an event are described by Kaplan-Meier and compared between groups by the log-rank test. The differences of the analysis variables are tested with the paired t-test or, in case of uneven distribution, the Wilcoxon-test (2-sided) for within group comparison. The Mann-Whitney U test will be used to detect differences between study groups. The following statistical values will be calculated where appropriate: mean, standard deviation, 95%-confidence interval of the mean, minimum, lower quartile, median, upper quartile, maximum, valid number, frequency count, and percentage. The laboratory values will be counted according to their normal ranges (below normal, normal, and above normal). Biometric analysis will be defined in the statistical analysis plan which has to be authorized before unblinding by the biometrician, the sponsor, and the principal investigator. One interim analysis will be performed after half of the patients have finished the clinical period for the primary endpoints.

All patient data (clinical and resource use) generated during the study will be recorded on the eCRFs specifically designed to meet the data recording requirements of the clinical study protocol provided by HealthEcon AG. All data management activities will be done according to ICH-GCP guidelines. Responsibility for data management is with HealthEcon AG, Basel, Switzerland. Throughout the study, all patient information in the eCRF will only be identifiable by means of an identification number (patient number) and possibly patient initials.

## Discussion

PEM is common in ESLD, with the prevalence ranging from 20% to 60% or even higher depending on the severity of liver insufficiency and the method of assessment. The prevalence and degree of PEM do not seem to relate to the etiology of liver disease per se [29]. In order to completely assess the nutritional status of the patients with ESLD, it is essential to obtain detailed information on body cell mass, biochemical function, and basal metabolism of the patients.

Muscle wasting is a major feature of malnutrition in ESLD [30], and is almost always present in patients waiting for LT [31]. Anthropometric evaluation of upper limbs is a simple, convenient, valuable, rapid, noninvasive, and inexpensive way to assess nutritional status, and it is easy to repeat during the follow-up period [32]. Combined anthropometric measurements of arm circumference and triceps skinfold thickness and calculation of arm muscle area are useful tools for the assessment of body muscle mass, which, together with fat mass, decrease in ESLD due to PEM and subsequent enhanced gluconeogenesis and protein breakdown. The decrease in muscle mass is greater than the reduction of body weight [33,34]. Also, mid-arm muscle circumference and handgrip strength measurements appear to be sensitive markers of body cell mass depletion [35] whereas triceps skinfold thickness provides a good clinical assessment of the fat body mass [36]. Urinary creatinine excretion correlates significantly with anthropometrically estimated muscle mass. However, it should be interpreted cautiously in the presence of coexisting renal dysfunction [37]. Although for a precise quantification of malnutrition in patients with ESLD, direct methods such as *in vivo *neutron activation analysis, dual-energy x-ray absorptiometry, or deuterium oxide dilution (to determine total body nitrogen, fat, and water, respectively) are preferred [38], these methods are expensive, not readily available, and therefore their use is not justifiable for a long-term clinical trial. Moreover, even the accuracy of such tests may be affected by fluid retention [3]. Laboratory tests can indicate the liver dysfunction, and the consequent biochemical derangements. Basal metabolism should be measured by indirect calorimetry, and if not available, may be calculated from Harris and Benedict's equation [39]. Ideal body weight may be accepted for calculation means [29]. Voluntary muscle function has been shown to be an independent predictor of outcome in alcoholic liver disease [40] and therefore may be useful as an objective tool to assess the response to nutrition in ESLD [41]. The "medical outcomes study short form 36" (SF-36) is a widely used and validated generic HRQL questionnaire [42-44].

In order to establish a nutritional support, an ideal product is what has undergone an extensive evaluation for safety and efficacy through prospective randomized clinical trials. Considerable evidence supports that IMPACT^® ^specialized nutrition support positively influences inflammatory, metabolic, and immune responses to major surgery [45]. However, any nutrient that can alter immune function deserves critical evaluation for potential adverse effects. Up to now, no adverse effects has been reported in patients supplemented with IMPACT^® ^[45]. Concerning the L-Arginine content of IMPACT^® ^and its use in patients with ESLD, a recent pilot study on fifteen patients with ESLD receiving two 74 g sachets of ORAL IMPACT^® ^(each containing 3.74 g L-Arginine) daily (twice as much as the daily administered amount in current study) for a median of 54 (range 10–168) days pre-transplant showed no adverse consequences that could be attributed to the nutritional product [23]. Furthermore, the total protein content of IMPACT^® ^is 16 g, which is far below the 1.2 g/kg daily protein requirement of the patients with ESLD [29].

## Conclusion

This randomized controlled trial is intended to investigate the long-term preoperative effects of ORAL IMPACT^® ^on the nutritional status as well as the outcome of the patients with ESLD listed for transplantation. The working hypothesis is that Oral IMPACT^® ^has the ability to downregulate the inflammatory responses to chronic disease and surgery through immune modulation. Safety and efficacy, anthropometric and physiological parameters, HRQL, as well as post-transplant morbidities and survival will be assessed.

## Abbreviations

ESLD- End-stage liver disease.

PEM- Protein-energy malnutrition.

HRQL- Health-related quality of life.

TNF- Tumor necrosis factor.

IL- Interleukin.

LTB5- Leukotriene B5.

PGE3- Prostaglandin E3.

PGI3- Prostaglandin I3.

TxA3- Thromboxane A3.

NO- Nitric oxide.

LT- Liver transplantation.

MELD- Model for end-stage liver disease.

eCRF- Electronic case report form.

BW- Body weight.

BMI- Body mass index.

MAMA- Mid-arm muscle area.

MAC- mid-arm circumference.

MAMC- Mid-arm muscle circumference.

TSF- Triceps skinfold thickness.

LBM- Lean body mass.

CHI- Creatinine height index.

ALT- Alanine aminotransferase.

AST- Aspartate aminotransferase.

INR- International normalized ratio.

GH- Growth hormone.

IGF-1- Insulin-like growth factor-1.

REE- Resting energy expenditure.

SF-36- Medical outcomes study short form 36.

CRP- C-reactive protein.

ICU- Intensive care unit.

PaCO_2- A_rterial pressure of carbon dioxide.

PaO_2_- Arterial pressure of oxygen.

FiO_2- _Fraction of inspired oxygen.

AE- Adverse event.

SAE- Serious adverse event.

ICH-GCP- International conference on harmonisation of technical requirements for registration of pharmaceuticals for human use (ICH): guidance on good clinical practice (GCP).

ANOVA- Analysis of variance.

## Competing interests

The author(s) declare that they have no competing interests.

## Authors' contributions

HS, PS and AN conceived and designed the study based on their preclinical and clinical results and experiences, developed essential study documents, and formulated the statistical analysis plan. HS and PS performed quality review to assure adherence to current guidelines and laws. JE, JS and MWB supported the design of the study with their knowledge and experience. PS conducts the study as the principal investigator. AN and PS wrote the manuscript. All authors read and approved the final manuscript.
